# Effect of epidural analgesia in trial of labor after cesarean on maternal and neonatal outcomes in China: a multicenter, prospective cohort study

**DOI:** 10.1186/s12884-019-2648-1

**Published:** 2019-12-16

**Authors:** Jing Sun, Xuetao Yan, Aiwu Yuan, Xiaolei Huang, Yuci Xiao, Liwei Zou, Danyong Liu, Ting Huang, Zhao Zheng, Yuantao Li

**Affiliations:** 1 Department of Anesthesiology, Affiliated Shenzhen Maternity & Child Healthcare Hospital, Southern Medical University, No.2004 Hongli Road, Futian District, Shenzhen, 518028 Guangdong China; 2 Department of Anesthesiology, Bao’an Maternal and Child Health Hospital, Jinan University, Shenzhen, 518100 China; 3 Department of Anesthesiology, Longgang District Maternity & Child Healthcare Hospital of Shenzhen City, Shenzhen, 518172 China; 4 Department of Obstetrics, Affiliated Shenzhen Maternity & Child Healthcare Hospital, Southern Medical University, Shenzhen, 518028 Guangdong China

**Keywords:** Epidural analgesia, Trial of labor after cesarean, Vaginal birth after cesarean, Maternal and neonatal outcomes, Propensity score matching

## Abstract

**Background:**

The trial of labor after cesarean section (TOLAC) is a relatively new technique in mainland of China, and epidural analgesia is one of the risk factors for uterine rupture. This study aimed to evaluate the effect of epidural analgesia on primary labor outcome [success rate of vaginal birth after cesarean (VBAC)], parturient complications and neonatal outcomes after TOLAC in Chinese multiparas based on a strictly uniform TOLAC indication, management and epidural protocol.

**Methods:**

A total of 423 multiparas undergoing TOLAC were enrolled in this study from January 2017 to February 2018. Multiparas were divided into two groups according to whether they received epidural analgesia (study group, *N* = 263) or not (control group, *N* = 160) during labor. Maternal delivery outcomes and neonatal characteristics were recorded and evaluated using univariate analysis, multivariable logistic regression and propensity score matching (PSM).

**Results:**

The success rate of VBAC was remarkably higher (85.55% vs. 69.38%, *p* < 0.01) in study group. Epidural analgesia significantly shortened initiating lactation period and declined Visual Analogue Score (VAS). It also showed more superiority in neonatal umbilical arterial blood pH value. After matching by PSM, multivariable logistic regression revealed that the correction of confounding factors including epidural analgesia, cervical Bishop score at admission and spontaneous onset of labor were still shown as promotion probability in study group (OR = 4.480, 1.360, and 10.188, respectively; 95%CI = 2.025–10.660, 1.113–1.673, and 2.875–48.418, respectively; *p* < 0.001, *p* = 0.003, and *p* < 0.001, respectively).

**Conclusions:**

Epidural analgesia could reduce labor pain, and no increased risk of postpartum bleeding or uterine rupture, as well as adverse effects in newborns were observed. The labor duration of multiparas was increased, but within acceptable range. In summary, epidural analgesia may be safe for both mother and neonate in the three studied hospitals.

**Trial registration:**

Chineses Clinical Trial Register, ChiCTR-ONC-17010654. Registered February 16th, 2017.

## Background

The proportion of pregnant women undergoing cesarean section has augmented steadily in the past few decades and reached the highest in the world, especially in China [1, 2]. Cesarean section can reduce maternal and neonatal mortality, but overuse may result in severe maternal outcomes, such as enhanced risk of death [3]. At present, delivery modes for multiparas receive increasing concerns with the implementation of the two-child policy in China. After the first cesarean section, the alternative mode of subsequent labor includes repeated cesarean section (RCS) and the trial of labor after cesarean (TOLAC). Recent data show that TOLAC is the most effective method of delivery because of the less expense and better effect in reducing the risk of postpartum hemorrhage (PPH) and pelvic adhesions [4]. In addition, several investigators had attempted to create formulae to calculate individual specific TOLAC results. Costantine MM et al. [5] established a predictive model for a cohort of women with a single previous cesarean section, and found that the success rate of vaginal birth after cesarean (VBAC) was 10–20% lower than that of women who predicted > 50%VBAC success rate. Grobman WA et al. [6] also created a nomogram using factors available at the first prenatal visit and developed a useful tool to measure patient-specific VBAC success rate. These studies indicate that multiparas with an expected success rate of > 50% are appropriate candidates for TOLAC, which needs to be clinically validated.

Epidural analgesia is a pre-requisite for many women to choose TOLAC, and for obstetricians, it is a means of allowing adequate and long-lasting pain relief in the management of normal and dysfunctional labors. However, none of above predictive models has mentioned analgesic effect. TOLAC is still in the early stages in mainland of China because of its unique medical environment. Notably, the doctor-patient relationship in China is tense due to patient distrust. Therefore, in order to reduce medical disputes, doctors often have to choose the surgical treatment according to patients’ needs, rather than the natural childbirth based on patients’ own conditions [7]. With increasing international acceptance of TOLAC and improved technology, more and more Chinese medical institutions begin to use TOLAC. It has been reported that the epidural analgesia is unsafe for maternal and newborn at risk of uterine rupture, but the 2010 American Association of Obstetricians and Gynecologists (ACOG) guidelines explicitly recommend that epidural anesthesia in TOLAC is safe [8, 9]. In addition, different policies for labor dystocia management and epidural anesthesia permissiveness at TOLAC challenge the interpretation of previous studies. Several reports have revealed the relationship between labor pain intensity and dystocia, suggesting that obstructed labor rate is higher as labor pain increased, while pain relief makes labor go smoothly [10–12]. A correlation was shown of endogenous plasma epinephrine and cortisol levels with labor progression [13, 14]. Neumark J et al. [15] have demonstrated that cortisol level significantly rises in women undergoing epidural analgesia, while decreases as epinephrine level declined after pain relief. Besides, the decrease in alpha- and beta-adrenergic stimulus may enhance uterine perfusion and further lead to an effective uterine contraction pattern, since its sensitivity is characteristic of the uteroplacental vascular bed rather than the systemic vasculature [16]. Therefore, we hypothesize that epidural analgesia reduces maternal epinephrine levels by eliminating psychological and physical stress associated with painful uterine contractions, thus promotes delivery. This brings more opportunities for TOLAC and emphasizes the low rate of side effects of epidural anesthesia for both mother and fetus [17].

At present, epidural analgesia has not been widely used in TOLAC due to controversy. Domestic and foreign studies on the application of epidural analgesia in TOLAC are mostly small samples or retrospective studies, but lack of large sample investigation or systematic study. This multi-center study aimed to evaluate the effect of epidural analgesia on primary labor outcome (success rate of VBAC), parturient complications and neonatal outcomes after TOLAC in Chinese multiparas based on a strictly uniform TOLAC indication, management, and epidural protocol.

## Methods

### Ethics and informed consent

This multi-center, prospective cohort study was approved by the human research committee of Affiliated Shenzhen Maternity & Child Healthcare Hospital, Bao’an Maternal and Child Health Hospital, and Longgang District Maternity & Child Healthcare Hospital of Shenzhen City, and written informed consents were obtained from all subjects participating in the trial. The study was registered prior to patient enrollment at the Chinese Clinical Trial Register, a participant in the World Health Organization International Clinical Trials Registry Platform (ChiCTR-ONC-17010654, Principal investigator: Jing Sun, Date of registration: February 16th, 2017).

### Study design and multiparas’ recruitment

**Fig. 1:**
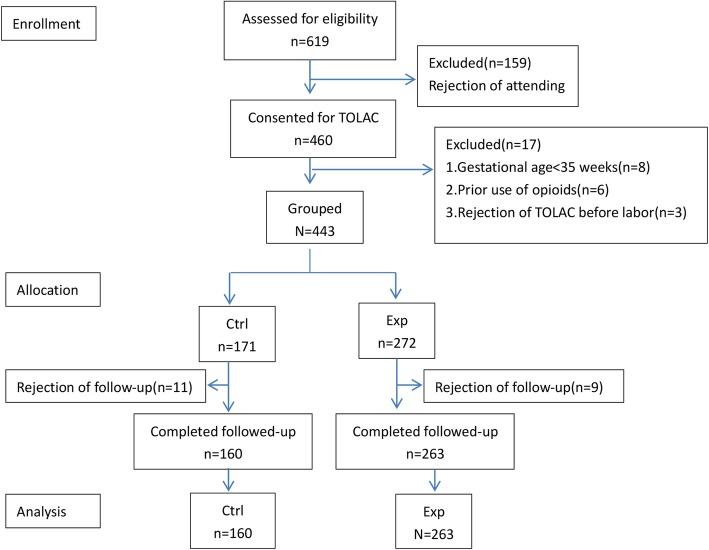
Flow chart of the study

Totally 443 multiparas undergoing TOLAC were enrolled in this study from January 2017 to February 2018. They were divided into two groups according to their own requests of epidural analgesia, i.e., multiparas who received epidural analgesia were in study group; while matched multiparas who did not receive epidural analgesia were in control group. After excluding 20 follow-up misses, a total of 423 multiparas, including 263 in study group and 160 in control group, were entered into the final analysis (Fig. 1), and the baseline maternal demographic and obstetric characteristics were recorded (Table 1). Inclusion criteria: multiparas with a single live fetus in cephalic presentation at > 35 weeks; no systemic analgesics were currently used; eligible for TOLAC under department admission and management protocol. The eligibility criteria for TOLAC (as assessed by obstetricians) were as follows: a. willingness of undergoing TOLAC and acceptance of the possible risks; b. good health condition without contraindications of vaginal delivery; c. immediate access to emergency surgery; d. confirmation of successful history of cesarean section with low-transverse segment, no delayed incision, scheduled recovery, as well as no late postpartum hemorrhage and postpartum infection; e. more than 1 year from last cesarean section; f. no history of uterine rupture; g. fetal weight < 4200 g (estimated through clinical assessment or ultrasound exam within a week from admission). TOLAC can be performed regardless of the number of uterine closure layers in cesarean section at the first time, and a diagnosis of dystocia of labor for previous cesarean is not considered as a contraindication for TOLAC. However, multiparas with a history of mental illness and contraindications to epidural labor analgesia were excluded from the study. All eligible women were informed of the study and signed a consent form when they entered Labor Analgesia Consultation Clinic during 34–35 weeks prenatal. However, it was also possible for a multipara to change her mind when she entered the delivery room and began delivery.

**Table 1:** Baseline Maternal Demographic and Obstetric Characteristics

Characteristic	TOLAC (control)		TOLAC (study)		Statistics	*P* value
	N (All)	N (%) or Mean ± SD	N (All)	N (%) or Mean ± SD		
Hospital name	160		263		41.02053	< 0.001
MCH		131(81.88%)		179(68.06%)		
BA		1(0.62%)		59(22.43%)		
LG		28(17.50%)		25(9.51%)		
General information						
Age (y)	160	32 ± 4	262	32 ± 4	21,481.5	0.667
Gestational age (day)	158	272 ± 14	260	274 ± 10	18,846.5	0.157
BMI (kg/m^2^)	158	26.45 ± 2.93	259	26.59 ± 3.08	19,040.5	0.234
Maternal education > 12 y	116	116(72.96%)	193	193(74.23%)		0.819
Husband education > 12 y	118	118(74.21%)	207	207(79.62%)		0.228
Housewives	159	13(8.18%)	259	21(8.11%)		1
History of pregnancy and childbirth						
History of previous vaginal delivery	151	14(9.27%)	254	15(5.91%)		0.233
Gravidity	157		260		2.264	0.322
2		70(44.59%)		98(37.69%)		
3		62(39.49%)		121(46.54%)		
> 3		25(15.92%)		41(15.77%)		
Time from last cesarean section (y)	156		261			0.042
≤ 3		1(0.64%)		9(3.45%)		
≤ 5		88(56.41%)		165(63.22%)		
> 5		67(42.95%)		87(33.33%)		
Progress of Labor during previous Caesarean section (cervical dilation: cm)	148		256		5.093	0.165
0		83(56.08%)		130(50.78%)		
< 3		36(24.32%)		87(33.98%)		
≥ 3		19(12.84%)		29(11.33%)		
10		10(6.76%)		10(3.91%)		
Cervical score at admission (score)	151	6.54 ± 1.76	253	6.22 ± 2.12	20,321.5	0.27
Cervical dilation at admission < 3 cm	156	108(69.23%)	259	237(91.51%)		< 0.001
Effacement ≤50	151	47(31.13%)	256	80(31.25%)		1
The onset of labor: Spontaneous	156	136(87.18%)	262	251(95.80%)		0.002
Oxytocin during labor	144	10(6.94%)	244	22(9.02%)		0.568
Maternal comorbidities						
Gestational diabetes mellitus	155	16(10.32%)	261	23(8.81%)		0.606
Hypertensive disorders of pregnancy	155	3(1.94%)	260	4(1.54%)		0.716
Hypothyroidism	155	4(2.58%)	260	8(3.08%)		1
Asthma	153	0(0.00%)	261	3(1.15%)		0.299
Prepartum Laboratory test						
HGB (g/L:115–150)	159	119.28 ± 15.05	259	116.68 ± 11.17	22,643	0.087
PLT (125–350)	159	209.47 ± 55.51	260	210.03 ± 53.70	20,399.5	0.822

*BMI* Body mass index, *HGB* Hemoglobin, *PLT* Platelet

Data are Mean ± SD, or N (%). *SD* Standard deviation

Comparisons were made using two-sided Student’s *t* test or Wilcoxon rank sum test for non-normally distributed variables

Comparisons were made using Pearson’s Chi-squared test and Fisher’s exact test for proportions

### The method of analgesia

All of the investigated multiparas were observed in the delivery room, and initial laboratory evaluation was performed including blood type, hemoglobin, complete platelet count and coagulation function. They underwent continuous electronic fetal monitoring throughout delivery and were carefully evaluated to assess TOLAC’s suitability. Any of the following indications should be noted when an immediate emergency cesarean section was happened, including cessation of labor, abnormal fetal heart rates (FHRs) and suspected uterine rupture. Multiparas admitted for TOLAC were managed by certified midwives who determined eligibility for TOLAC, induction of labor or oxytocin augmentation, mode of delivery, use of episiotomy, forceps and vacuum extraction for operative vaginal delivery in accordance with maternal and fetal indications made by board certified obstetricians. The multiparas participating in the study did not experience changes in obstetric clinical management during delivery.

After entering the delivery room, multiparas in the study group were placed in the left lateral position with opened peripheral venous access. Then, epidural puncture (AS-E epidural puncture package) was performed through L2-L3 or L3-L4 intervals, and the epidural catheter was inserted at a depth of 3–4 cm. Then, an experimental dose (a total of 3 mL of 1:200,000 adrenalin+ 1.5% lidocaine) was injected. If there was no local anesthetic poisoning or other abnormal reactions, the catheter was fixed and the multiparas were placed in the supine position. For pain relief, 10 mL of 0.1% ropivacaine mixed solution was injected once through the epidural catheter. If there were no obvious adverse reactions such as hypotension, nausea and vomiting or local anesthetic poisoning symptoms after 30 min of observation, an analgesia pump (ZZB-I impulse type, 200 mL) was connected, with a ready to use solution of 0.08% ropivacaine and sufentanil 0.4 μg/ml. Parameter setting: pulse frequency once/h, dose 10 mL, infusion rate 400 ml/h, PCA dose 8 ml, locking time 30 min. Epidural labor analgesia continued until the baby was delivered. Multiparas could terminal their epidural infusion at the request of the obstetric care provider for clinical indications. There was no standardization of the indication for termination nor was there a specific requirement other than obstetric request. Blood pressure was measured every 5 min during the first 20 min and hourly during the continuous of patient-controlled analgesia usage [18]. Meanwhile, Visual analog scale (VAS) pain scores was recorded 30 min after the epidural loading dose.

### Observational index

The primary outcome was the success rate of VBAC. Maternal and neonatal parameters were recorded. The maternal parameters included demographic characteristics, mode of delivery, reason for cesarean, uterine rupture, postpartum hemorrhage (a total blood loss > 500 ml within 24 h), labor duration, initiating lactation period (from fetal delivery to maternal conscious breast swelling, and the milk overflow on light pressure), and VAS score. VAS score was used to assess the pain level which presented a 10-cm unmarked line with endpoints labeled “no pain” and “worst pain imaginable” to let patients mark their pain level on [19].

Neonatal parameters were assessed by Apgar score at 1 min and 5 min, birth weight, umbilical arterial blood pH value and fetal distress (category III FHR tracing, meconium amniotic fluid with an abnormal FHR, or an umbilical cord blood pH < 7.2).

### Statistical analysis

All data were entered and analyzed in R software (version 3.5.0). Continuous data were expressed as Mean ± standard deviation (x ± s) and *t*-test was used for comparison between groups. Measurement data not conforming to normal distribution were compared using two-sided Student’s T test or Wilcoxon rank sum test. Categorical variables were presented as frequencies and percentages; Fisher’s exact test or Pearson’s Chi-squared test was used to examine difference between the groups. The Shapiro-Wilk test was used to assess the normal distribution of the data. Univariate analysis was used to analyze all risk factors that may affect outcomes of this study. Multivariable logistic regression models were adjusted to examine the effect of epidural analgesia on outcomes in TOLAC. Propensity score matching (PSM) were used in this study to reduce the potential selection bias and validate the results again. *P* value < 0.05 was considered the statistical significance.

## Results

### Baseline maternal demographic and obstetric characteristics

The study flow chart was shown in Fig. 1. Totally 423 multiparas enrolled into the final analysis were divided into two groups according to whether they received epidural analgesia (study group, *n* = 263) or not (control group, *n* = 160) during labor. There were no significant differences in age, BMI, history of previous vaginal delivery, gravidity, cervical Bishop score at admission, effacement <=50, oxytocin during labor, maternal comorbidities and prepartum laboratory test between the two groups. However, the factors including hospitals, time from last cesarean section, cervical dilation at admission < 3 cm, and spontaneous onset of labor in study group were notably different from those in control group (*p* < 0.05) (see Table 1). Although these baseline factors were statistically different, subsequent univariate analysis, multivariate logistic regression and PSM may eliminate the effect of these confounding factors.

### Primary outcome of maternal delivery in TOLAC

During the childbirth, there were two types of delivery according to multipara’s status. As shown in Table 2, the VBAC rate was significantly higher in the study group than that in control group (85.55% vs. 69.38%, *p* < 0.001). According to our coding, there was a higher rate of repeat cesarean sections in non-analgesia group than analgesia group [(49/160, 30.63%) vs. (38/263, 14.45%), *p* = 0.00153] due to fetal distress, stagnation of labor, fever or intrauterine infection, threatened uterine infection, unbearable pain, and change in fetal station.

**Table 2:** Primary outcome of Maternal Delivery in TOLAC

Characteristics	TOLAC (control)		TOLAC (study)		*P* value
	N (All)	N (%) or Mean ± SD	N (All)	N (%) or Mean ± SD	
Mode of delivery	160		263		< 0.001
VBAC		111(69.38%)		225(85.55%)	
Cesarean		49(30.63%)		38(14.45%)	
Reason for cesarean	49		38		0.00153
Fetal distress		10		13	
Stagnation of labor		6		7	
Fever or intrauterine infection		5		13	
Threatened uterine rupture		3		2	
Unbearable pain		23		4	
Change in fetal station		2		3	

*VBAC* Vaginal birth after cesarean section

Data are mean ± SD, or n (%). *SD* Standard deviation

Comparisons were made using two-sided Student’s t test or Wilcoxon rank sum test for non-normally distributed variables

Comparisons were made using Pearson’s Chi-squared test and Fisher’s exact test for proportions

### Secondary outcomes of maternal delivery in TOLAC

For safety reasons, the hospital had established stringent non-labor induction standards and strictly controlled the use of oxytocin in TOLAC. So labor of multipara was mostly spontaneous. Each woman went through three stages during labor. From Table 3, the labor durations in study group were obviously lower than those in control group in the first and second stages of labor (*p* < 0.001), while the labor duration between the two group in the third stage of labor was similar (*p* = 0.294). Instrument-assisted delivery could be used when delivery was too long. In women who had a successful VBAC, the rate of instrumental delivery was 16.5% (70/423), with 16.8% (27/160) in control group and 16.3% (43/263) in study group, indicating no significant difference (*p* = 1). There were no differences in uterine rupture, PPH, perineum or cervical laceration between the two groups. In addition, the occurrence of episiotomy was higher for multiparas with epidural than that without epidural (43.36% vs. 28.19%, *p* < 0.05), and the initiating lactation period was delayed in control group (7 ± 12 h vs. 11 ± 13 h, *p* < 0.001). At the beginning, the mean VAS score of epidural pain in control group was notably lower than that in study group (6.34 ± 2.16 vs. 8.20 ± 1.19, *p* < 0.001). Thirty minutes later, the mean VAS score for epidural analgesia group fell from 8.20 ± 1.19 to 0.94 ± 1.61, while for non-epidural analgesia group, the mean VAS score increased from 6.34 ± 2.16 to 7.00 ± 2.16. When the uterus expanded to 6 cm and 10 cm, the mean VAS pain scores in study group were significantly lower than those in control group [(1.09 ± 1.71) vs. (7.67 ± 2.33); (1.78 ± 2.00) vs. (8.64 ± 2.13), *p* < 0.001]. However, when the immediate delivery of fetus, the mean VAS pain scores were reversed in the two groups (*p* < 0.002).

**Table 3:** Secondary Outcomes of Maternal Delivery in TOLAC

Secondary outcomes	TOLAC (control)		TOLAC (study)		Statistics	*P* value
	N (All)	N (%) or Mean ± SD	N (All)	N (%) or Mean ± SD		
Initiating lactation period (h)	157	11 ± 13	262	7 ± 12	24,276.5	0.002
Uterine rupture	160	0(0%)	263	0(0%)		1
PPH	154		260			1
<=500		151(98.05%)		255(98.08%)		
> 500		3(1.95%)		5(1.92%)		
Episiotomy	149	42(28.19%)	256	111(43.36%)		0.003
Perineum/Cervical laceration	149	70(46.98%)	255	106(41.57%)		0.3
Instrumental delivery in VBAC	27		43			1
Vacuum extraction		15(13.51%)		23(10.22%)		
Forceps		12(10.81%)		20(8.89%)		
Labor duration in VBAC						
The first labor duration (min)	108	334.14 ± 225.94	221	526.93 ± 266.85	6050.5	< 0.001
The second labor duration (min)	102	28.09 ± 31.62	217	46.14 ± 32.64	5836	< 0.001
The third labor duration (min)	108	8.62 ± 3.87	222	9.43 ± 5.03	11,211.5	0.294
VAS pain at epidural						
T0: time 0	99	6.34 ± 2.16	219	8.20 ± 1.19	5191.5	< 0.001
T1: PCEA after 30 min	100	7.00 ± 2.16	217	0.94 ± 1.61	21,103	< 0.001
T2: cervical: 6 cm	100	7.67 ± 2.33	211	1.09 ± 1.71	20,404.5	< 0.001
T3: cervical: 10 cm	99	8.64 ± 2.13	210	1.78 ± 2.00	20,213	< 0.001
T4: immediate delivery of the fetus	97	1.65 ± 2.44	209	1.88 ± 1.60	8062	0.002

*PPH* Postpartum hemorrhage, *VAS* Visual analog scale, *VBAC* Vaginal birth after cesarean section

Data are mean ± SD, or n (%). *SD* Standard deviation

Comparisons were made using two-sided Student’s t test or Wilcoxon rank sum test for non-normally distributed variables

Comparisons were made using Pearson’s Chi-squared test and Fisher’s exact test for proportions

### Neonatal outcomes in TOLAC

Neonates in both groups were of similar weight and Apgar scores. The limitation of Apgar score may cause misdiagnosis and missed diagnosis of neonatal asphyxia. To make up for the deficiency, umbilical arterial blood pH value was also adopted in the diagnosis of neonatal asphyxia [20]. As shown in Table 4, the number of neonates whose pH value was lower than 7.2 in control group was remarkably higher than that in study group, while the number of neonates with pH > 7.2 was reversed (*p* = 0.001). The normal PCO2 score is between 35 and 45. It was obvious that the score in control group was above normal, which was significantly higher than that in study group (46.50 ± 10.54 vs. 43.80 ± 10.44, *p* = 0.041). In addition, the base excess required by the study group was significant less than the control group (*p* < 0.001). These results suggested that the physical conditions of neonates born to multipara who received epidural analgesia were better.

**Table 4:** Neonatal Outcomes in TOLAC

Characteristic	TOLAC (control)		TOLAC (study)		Statistics	*P* value
	N (All)	N (%) or Mean ± SD	N (All)	N (%) or Mean ± SD		
Neonatal results						
Weight	148		250			0.903
< 3500		114(77.03%)		190(76.00%)		
≥ 3500		34(22.97%)		60(24.00%)		
Admission to neonatal ward after birth	153	12(7.84%)	256	20(7.81%)		1
1 min Apgar	159	0(IRQ)	261	0(IRQ)	12,485.5	0.535
5 min Apgar	159	0(IRQ)	261	0(IRQ)	12,542	0.292
Umbilical arterial blood						
PH	119		220			0.001
< 7.2		20(16.81%)		12(5.45%)		
> =7.2		99(83.19%)		208(94.55%)		
PCO2 (mmHg)	118	46.50 ± 10.54	220	43.80 ± 10.44	14,729	0.041
Base excess (BE. mmol. L)	120	−6.04 ± 3.06	220	−4.38 ± 3.00	8989	< 0.001

*IQR* Interquartile range

Data are Mean ± SD, or n (%)

Comparisons were made using two-sided Student’s t test or Wilcoxon rank sum test for non-normally distributed variables

Comparisons were made using Pearson’s Chi-squared test and Fisher’s exact test for proportions

### Univariate and multivariate analysis of mode of delivery in TOLAC

Mode of delivery of multiparas in TOLAC was the dependent variable, epidural analgesia and other confounding factors were the independent variables. The univariate analysis in Table 5 showed that epidural analgesia, hospital, age, cervical dilation, cervical score at admission, effacement, the onset of labor, progress of labor in previous caesarean section (cervical dilation: < 3 cm) and neonatal weight were associated with mode of delivery in TOLAC. However, it was not clear whether these indices play an independent role in the mode of delivery or act in combination with other factors. Thus, we analyzed the above indices with multivariate logistic regression. The results revealed that the correction of confounding factors including epidural analgesia, hospital, age, cervical Bishop score at admission, spontaneous onset of labor, and neonatal weight were still shown as promotion probability in epidural analgesia group (OR = 2.590, 0.138, 0.853, 1.421, 6.801 and 0.360, respectively; 95%CI = 1.315–5.165, 0.046–0.391, 0.774–0.933, 1.177–1.727, 2.093–23.518 and 0.183–0.711, respectively; *p* = 0.006, *p* < 0.001, *p* < 0.001, *p* < 0.001, *p* = 0.002 and *p* = 0.003, respectively). The multivariate logistic regression model was proved well fitness by Hosmer and Lemeshow goodness of fit (GOF) test (*p* = 0.43), and the expansion factor of each factor was VIF < 2.

**Table 5:** Univariate and Multivariate analysis of Mode of delivery in TOLAC

Variable	Univariate		Multivariate(*n* = 354)	
	*P*	OR (95%CI)	*P*	OR (95%CI)
Epidural analgesia	< 0.001	2.614(1.620–4.248)	0.006	2.590(1.315–5.165)
Hospital name				
MCH		1(reference)		1(reference)
BA	0.473	1.340(0.623–3.197)	0.141	2.253(0.799–7.068)
LG	< 0.001	0.214(0.115–0.396)	< 0.001	0.138(0.046–0.391)
General information				
Age (y)	0.015	0.921(0.861–0.983)	< 0.001	0.853(0.774–0.933)
Gestational age (day)	0.421	0.990(0.965–1.011)		
BMI (kg/m^2^)	0.129	0.944(0.875–1.018)		
Maternal education > 12 y	0.187	0.681(0.375–1.184)		
Husband education > 12 y	0.114	0.604(0.312–1.099)		
Housewives	0.193	2.036(0.776–7.000)		
History of pregnancy and childbirth				
History of previous vaginal delivery	0.615	0.796(0.343–2.076)		
Gravidity time (times)				
2		1 (reference)		
3	0.78	1.076(0.641–1.804)		
> 3	0.971	1.013(0.514–2.083)		
Time from last cesarean section (year)				
≤ 3		1(reference)		
3–5	0.246	2.282(0.477–8.592)		
> 5	0.923	1.071(0.223–4.046)		
Progress of Labor in previous caesarean section (cervical dilation: cm)				
0		1(reference)		
< 3	0.029	1.882(1.083–3.381)		
≥ 3	0.148	1.827(0.844–4.417)		
10	0.062	6.942(1.391–126.155)		
Cervical score at admission (score)	0.003	1.203(1.066–1.360)	< 0.001	1.421(1.177–1.727)
Cervical dilation at admission (cm) ≥3	0.021	2.629(1.232–6.513)	0.058	2.698(1.026–8.200)
Effacement	0.027	1.750(1.062–2.865)		
The onset of labor: Spontaneous	< 0.001	7.428(3.484–16.431)	0.002	6.801(2.093–23.518)
Oxytocin during labor	0.044	0.451(0.211–1.011)		
HGB (g/L:115–150)	0.843	0.998(0.981–1.018)		
PLT (125–350)	0.924	1.000(0.996–1.005)		
Labor duration				
The first labor duration (min)	0.553	1.003(0.996–1.018)		
The second labor duration (min)	0.37	1.157(0.983–1.945)		
The third labor duration (min)	0.859	0.970(0.771–1.779)		
Neonatal				
Weight (g) ≥3500	0.015	0.512(0.301–0.884)	0.003	0.360(0.183–0.711)

*OR* Odds ratio, *CI* Confidence interval;

Multivariable logistic regression included entered factors variable as *p* < 0.05 (Epidural analgesia, Hospital, Age, Progress of Labor in previous caesarean section, Cervical score at admission, cervical dilation at admission, effacement, the onset of labor: Spontaneous, Oxytocin during labor, Neonatal Weight) and using stepwise regression by AIC criteria. Hosmer-Lemeshow goodness of fit (GOF) test: Chi-squared = 8.034, df = 8, *P* value = 0.430 McFadden’s pseudo-R squared =0.249 Cox & Snell pseudo-R squared = 0.223 Nagelkerke pseudo-R squared =0.350. All VIFs < 2

### Univariate and multivariate analysis of mode of delivery in TOLAC matched by PSM

Furthermore, PSM was used to reduce the potential selection bias and validate the results again. We used 1:1 nearest neighbor matching method to select 118 multiparas in study group (96 unmatched) and 118 multiparas with confounding factors in control group (see Table 6).

**Table 6:** Univariate and Multivariate analysis of Mode of delivery in TOLAC matched by PSM

Variable	Univariate(*n* = 236)		Multivariate(n = 236)	
	*P*	OR (95%CI)	*P*	OR (95%CI)
Epidural analgesia	< 0.001	4.361(2.202–9.198)	< 0.001	4.480(2.025–10.660)
Hospital name				
MCH		1(reference)		
BA	0.608	1.265(0.539–3.331)		
LG	0.984	0.000(NA-13382569087647956.000)		
General information				
Age (y)	0.892	1.006(0.923–1.095)		
Gestational age (day)	0.443	0.987(0.952–1.014)		
BMI (kg/m^2^)	0.211	0.940(0.851–1.038)		
Maternal education > 12 y	0.078	0.494(0.213–1.042)		
Husband education > 12 y	0.283	0.648(0.278–1.380)		
Housewives	0.523	1.515(0.480–6.705)		
History of pregnancy and childbirth				
History of previous vaginal delivery	0.203	2.641(0.728–16.976)		
Gravidity time (times)				
2		1(reference)		
3	0.462	0.776(0.389–1.511)		
> 3	0.237	2.027(0.682–7.493)		
Time from last cesarean section (year)				
≤ 3		1(reference)		
3–5	0.188	2.723(0.532–11.823)		
> 5	0.496	1.691(0.325–7.484)		
Progress of Labor in previous caesarean section (cervical dilation: cm)				
0		1(reference)		1(reference)
< 3	0.013	2.601(1.260–5.731)	0.168	1.811(0.794–4.361)
≥ 3	0.462	1.451(0.565–4.235)	0.892	0.927(0.320–2.974)
10	0.988	17,640,531.947(0.000-NA)	0.988	9,284,640.473(0.000-NA)
Cervical score at admission (score)	0.004	1.269(1.082–1.494)	0.003	1.360(1.113–1.673)
Cervical dilation at admission (cm) ≥3	0.068	2.742(1.028–9.522)		
Effacement	0.927	1.034(0.493–2.069)		
The onset of labor: Spontaneous	< 0.001	20.754(6.329–93.768)	< 0.001	10.188(2.875–48.418)
Oxytocin during labor	0.487	0.719(0.295–1.939)		
HGB (g/L:115–150)	0.592	0.994(0.973–1.017)		
PLT (125–350)	0.62	0.999(0.993–1.004)		
Neonatal				
Weight(g) ≥3500	0.304	0.682(0.334–1.452)		

*OR* Odds ratio, *CI* Confidence interval, *PSM* Propensity score matching

Data had been matched by using propensity score matching with 1:1 nearest neighbor matching. Multivariable logistic regression after PSM included entered factors variable as *p* < 0.05 (Epidural analgesia, Progress of Labor in previous caesarean section, Cervical score at admission, the onset of labor: Spontaneous) and using stepwise regression by AIC criteria. Hosmer-Lemeshow goodness of fit (GOF) test: Chi-squared = 13.627, df = 8, *P* value = 0.092 McFadden’s pseudo-R squared =0.232 Cox & Snell pseudo-R squared = 0.215 Nagelkerke pseudo-R squared =0.332. All VIFs < 2

**Fig. 2:**
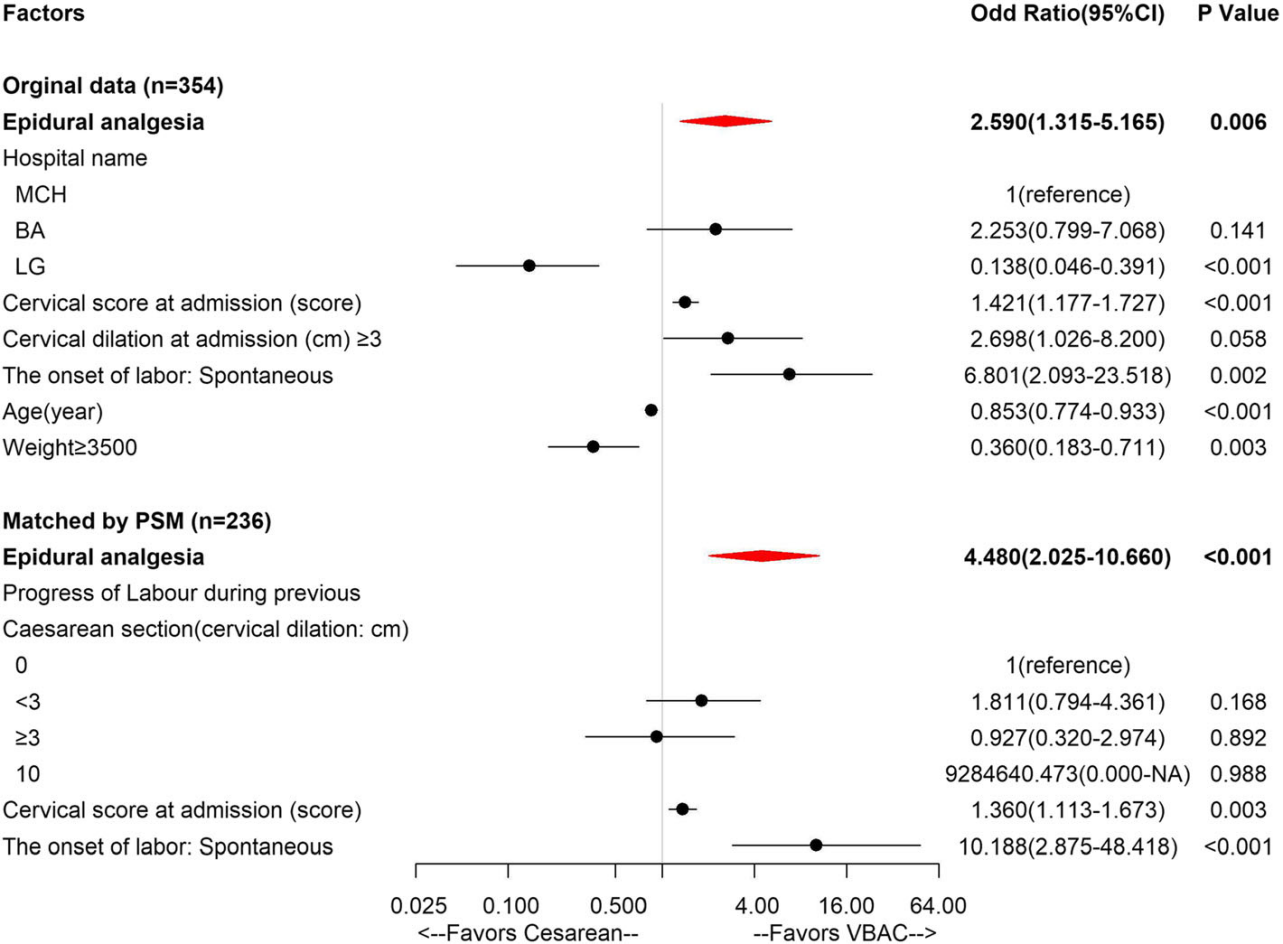
ROC of univariate and multivariate analysis of mode of delivery in TOLAC matched by PSM

After matching, the univariate analysis showed that epidural analgesia was still the promotion factor of spontaneous labor [OR = 4.361 (2.202–9.198), *p* < 0.001]. In addition, multivariate logistic regression model after the stepwise regression revealed that the correction of confounding factors including epidural analgesia, cervical Bishop score at admission and spontaneous onset of labor were still shown as promotion probability in study group (OR = 4.480, 1.360, and 10.188, respectively; 95%CI = 2.025–10.660, 1.113–1.673, and 2.875–48.418, respectively; *p* < 0.001, *p* = 0.003, and *p* < 0.001, respectively) (Fig. 2). The multivariate logistic regression model was proved well fitness by Hosmer-Lemeshow goodness of fit (GOF) test (*p* = 0.092), and the expansion factor of each factor was VIF < 2.

**Fig. 3:**
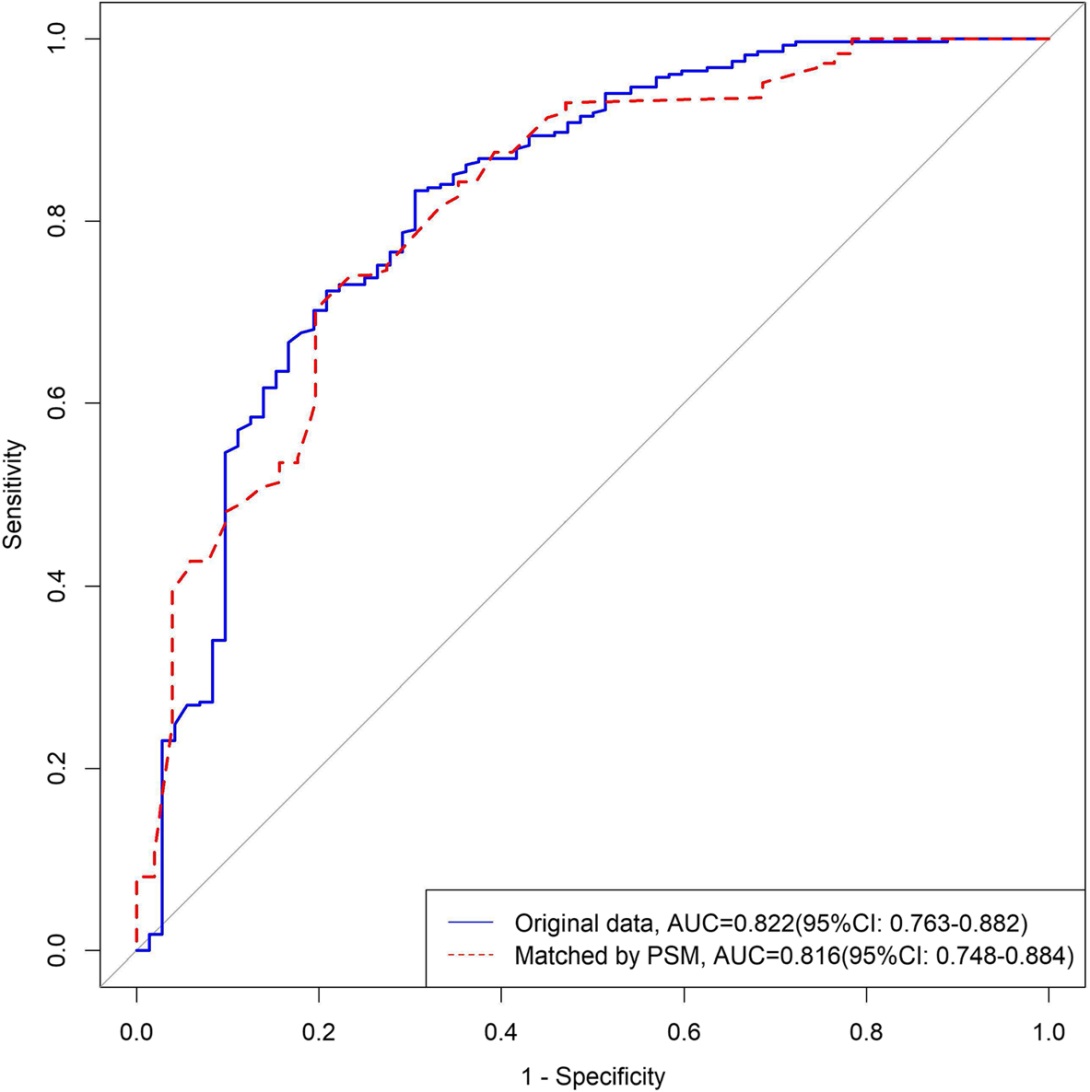
Two multivariate logistic regression was performed to predict the VBAC in TOLAC

As shown in Fig. 3, the two multivariate logistic regression was performed to predict the VBAC in TOLAC. The model without PSM showed that the area under the curve (AUC) was 0.822 (95%CI = 0.763–0.882) with sensitivity of 0.833, specificity of 0.694, positive predictive value (PPV) of 0.914, and negative predictive value (NPV) of 0.515. The model with PSM showed that AUC was 0.816 (95%CI = 0.7481–0.8841) with sensitivity of 0.702, specificity of 0.804, PPV of 0.929, and NPV of 0.427.

## Discussion

Labor is a painful experience, and about 30% of mothers have found it more painful than expected [21]. The previous cesarean section increases maternal emotional stress and affect their initial consents to TOLAC, while subsequent VBAC increases maternal satisfaction and motivation for future vaginal delivery [22]. In this study, we found that epidural analgesia remarkably increased the success rate of VBAC after TOLAC, and revealed some critical protective factors including cervical Bishop score at admission, spontaneous onset of labor, which showed that probability of VBAC with epidural analgesia was 2.027 times as non-epidural after TOLAC. Oxytocin was used in patients with weak contractions during labor, and no uterine rupture was found. However, most cases of vaginal trial were mainly normal labor. Lower acceptance and lack of experience of TOLAC in the mainland of China result in strict inclusion criteria for TOLAC eligibility might be the reason. This study also found that cervical conditions during delivery (Bishop score) had a positive correlation with vaginal delivery success rate. Consistently, Smith GCS et al. have clarified that parturients whose cervix is more than 4 cm and the cervical canal opens more than 25% in TOLAC received higher success rate [23].

The effect of multiparas’ age on TOLAC has been analyzed in many studies, but no uniform results. The study conducted by Regan, J et al. [24] have revealed that maternal age is not associated with the success or failure of VBAC in low-risk women. However, Minsart et al. [25] have proposed that when multivariate models were included, the independent factor associated with TOLAC was maternal age < 35 years. Moreover, Sentilhes et al. [8] have indicated that TOLAC’s failure rate increases as pregnant women age. Smith have also reported that with a 5-year old increase, the OR was adjusted of vaginal trial failure 1.22, 95% CI: 1.16–1.28, which was in line with our research [23].

Breast milk contains essential nutrients for the growth and development of infants. However, the pain during labor may delay lactogenesis [26]. Thus, initiating lactation period was a necessary index evaluating the effect of epidural analgesia. In the present study, the initiating lactation period was shorter in epidural group than non-epidural group. It was demonstrated that early or late initiation of epidural analgesia for labor had similar effects on all measured outcomes, which was inconsistent with our results regarding the initiating lactation period [27–29]. Previous study has indicated that the timely and effective analgesia after delivery helps the multipara to take the comfortable position so that newborn could suck nipple frequently, which was conducive to milk secretion [30].

Episiotomy is not supposed to be performed routinely, whereas it is inevitably required in many cases of operative vaginal delivery [31]. It is reported that episiotomy occurs in 65.8% of cases undergoing operative vaginal delivery [32]. In the present study, episiotomy usage rate was 43.36% in epidural group which was higher than 28.19% in non-epidural group, and the mild prolongation of labor did not increase the adverse effects of maternal and neonatal, no uterine rupture occurred as well. It is well known that reduction in uteroplacental perfusion conferred by uterine contractions during labor can increase the risk of asphyxia, neurological injury and death of fetuses. Moreover, long duration of labor may increase the risk of uterine rupture. Hence, long duration of labor should be avoided during VBAC through shortening the second stage of labor by operative vaginal delivery, further reducing the incidence of fetal distress [33]. Notably, a natural experiment conducted by J. Zhang et al. [34] has demonstrated that the second stage of labor is significantly longer by about 25 min, which is consistent with this study. The reason may be that during the second stage of labor, in addition to uterine contraction, pelvic floor muscles, active breath holding and other functions of multiparas are also needed, while epidural analgesia weakens the function of pelvic floor muscles and increases abnormal fetal position after delivery [35].

The overall epidural analgesia rate in this study was 62.17%. Among them, the Affiliated Shenzhen Maternity & Child Healthcare Hospital was 57.74%, the Bao’an Maternal and Child Health Hospital was 98%, and the Longgang District Maternity & Child Healthcare Hospital of Shenzhen City was 47.17%. The high rate of epidural analgesia in Bao’an Maternal and Child Health Hospital is mainly due to its comprehensive promotion of “painless hospital” construction, which is in response to the call of the world health organization “improve the painless delivery rate”. As long as there is no contraindication for intravertebral labor analgesia, all parturients could perform painless delivery. If a parturient refuse to give birth painlessly, she will have to go to another hospital, which reflects the differences between culture in Eastern and Western.

There are several limitations in this study. First, the multi-center study enriched the sample size, but different labor modes and labor analgesia management might influence the incidence of epidural analgesia and the high incidence of episiotomy. Notably, some midwives performed an active lateral incision to prevent tearing of the vulva. Therefore, there were some subjective factors in the implementation of lateral incision. Further experimental design should fully plan the surgery process, so that all operations are strictly unified. Second, the analyzed multiparas in this study were of the same ethnicity. There was a significant difference in the proportion of patients with painless labor in the three hospitals, indicating that the three hospitals had some selection bias in the patients with painless labor. In the follow-up process, multivariate logistic regression and PSM were performed to correct the impact of inter-hospital and other confounding factors. However, due to the sample size, the problem of selection bias of the previous sample itself cannot be completely eliminated (the coverage area of the hospital was different). Hence, the sample size needs to be further increased in the future to further verify this result. Third, observational cohort studies do not yield causality and can only see correlation analysis.

## Conclusions

Epidural analgesia could reduce labor pain, and no increased risk of postpartum bleeding or uterine rupture, as well as adverse effects in newborns were observed. The labor duration of multiparas was increased, but within acceptable range. In summary, epidural analgesia may be safe for both mother and neonate in the three studied hospitals. The data of present study can inform clinical delivery practice. However, in their provision of care medical practitioners should specifically assess the desires and expectations of the laboring women.

## Data Availability

The datasets compiled during the current study are available from the corresponding author on reasonable request.

## Abbreviations

ACOG: American Association of Obstetricians and Gynecologists
AUC: Area under the curve
FHRs: Abnormal fetal heart rates
GOF: Goodness of fit
NPV: Negative predictive value
PPH: Postpartum hemorrhage
PPV: Positive predictive value
PSM: Propensity score matching
RCS: Repeated cesarean section
TOLAC: The trial of labor after cesarean section
VAS: Visual Analogue Score
VBAC: Vaginal birth after cesarean
